# The efficacy of drug-eluting bead or conventional transarterial chemoembolization plus apatinib for hepatocellular carcinoma with portal vein tumor thrombus

**DOI:** 10.1038/s41598-022-09609-8

**Published:** 2022-04-06

**Authors:** Weihua Zhang, Linxia Wu, Lei Chen, Tao Sun, Yanqiao Ren, Bo Sun, Licheng Zhu, Ping Han, Chuansheng Zheng

**Affiliations:** 1 Department of Radiology, Union Hospital, Tongji Medical College, Huazhong University of Science and Technology, Wuhan, 430022 China; 2 Department of Interventional Radiology, Union Hospital, Tongji Medical College, Huazhong University of Science and Technology, Wuhan, 430022 China

**Keywords:** Cancer, Oncology

## Abstract

Transarterial chemoembolization (TACE) combined with apatinib has been used for advanced hepatocellular carcinoma (HCC), and the efficacy is good. The study was conducted to compare the efficacy and safety of drug-eluting bead TACE plus apatinib (D-TACE-A) with conventional TACE plus apatinib (C-TACE-A) in the treatment of HCC with portal vein tumor thrombus (PVTT). A total of 130 continuous patients who received D-TACE-A or C-TACE-A were included in the study from January 2017 to June 2020. Propensity score matching (PSM) was used to reduce potential selection bias. Before PSM, the median overall survival (mOS) (14 months) and median progression-free survival (mPFS) (7 months) in the C-TACE-A group were longer than the mOS (9 months; P = 0.001) and mPFS (4 months; P = 0.001) in the D-TACE-A group. After PSM, the mOS (14 months vs 9 months; P = 0.039) and mPFS (7 months vs 5 months; P = 0.009) in the C-TACE-A group were longer than those in the D-TACE-A group. In the multivariate regression analysis, C-TACE-A reduced the mortality rate and tumor progression rate compared with D-TACE-A. For the subgroup analysis, patients with VP1–2, without extrahepatic metastases, and with multiple TACE sessions who received C-TACE-A had a lower death risk and tumor progression risk than patients who received D-TACE-A. Before PSM, there was no statistically significant difference in any grade or grade III/IV adverse events (all P > 0.05). C-TACE-A could prolong mOS and mPFS in patients with PVTT, especially for patients with VP1–2 stage PVTT, no extrahepatic tumor metastases, and multiple TACE sessions.

## Introduction

Hepatocellular carcinoma (HCC) is one of the most common and lethal cancers worldwide. In 2020, 905 677 new HCC cases were diagnosed and 830 180 patients died from HCC worldwide^1^. The most commonly used classification for HCC is the Barcelona Clinic Liver Cancer (BCLC) stage^2^. For patients with early HCC (BCLC stage A), the European Association for the Study of the Liver (EASL) guidelines recommend radical treatments (transplantation, liver resection, or ablation) as the first-line treatments^3^. However, most patients are at an intermediate or advanced stage when they are diagnosed with HCC. For patients with advanced HCC, sorafenib and lenvatinib are recommended as their first-line choice (the median survival time of patients from sorafenib or Lenvatinib were 10.7 months and 13.6 months)^4,5^. However, for advanced HCC patients with portal vein tumor thrombus (PVTT), the survival of patients who receive sorafenib is not satisfactory. Thus, combination treatments are used in the treatment of HCC patients with PVTT. Previous studies have shown that transarterial chemoembolization (TACE) combined with sorafenib could prolong the survival of HCC patients with PVTT^6–9^. However, sorafenib is so expensive that many patients with advanced HCC cannot afford it, and the low response of sorafenib in the treatment of patients with advanced HCC has limited its usage in some advanced HCC patients. Thus, another selective drug, apatinib, is now used in the treatment of advanced HCC.

Some studies have shown that patients with advanced HCC can obtain more survival benefits from TACE plus apatinib than TACE alone^10–12^. However, two kinds of TACE (conventional TACE, C-TACE; and drug-eluting bead TACE, D-TACE) techniques are being used in the treatment of HCC. Previous high-quality studies have shown that patients with unresectable HCC who received D-TACE could obtain comparable survival benefits to those who received C-TACE, but D-TACE can reduce the pain of patients after the procedure^13–16^. However, there are few studies comparing the efficacy and safety of HCC patients receiving C-TACE plus other treatments with patients receiving D-TACE plus other treatments. Whether the survival benefits of HCC patients with PVTT who received D-TACE plus apatinib (D-TACE-A) are comparable with those of patients who received C-TACE plus apatinib (C-TACE-A) is still unclear. Few studies have focused on the combination treatments of C-TACE-A or D-TACE-A for the treatment of HCC patients with PVTT.

Thus, this study was conducted to compare the efficacy and safety of HCC patients with PVTT who received C-TACE-A with patients who received D-TACE-A.

## Materials and methods

### Patient selection

This retrospective study reviewed data from 385 patients receiving TACE-A from January 2017 to June 2020. After inclusion criteria and exclusion criteria, a total of 130 patients were included in the study. This study was approved by the Ethics Committee Board of Tongji Medical College, Huazhong University of Science and Technology, and informed consent was waived because this is a retrospective study.

The inclusion criteria were as follows: (1) patients were diagnosed with HCC with PVTT by CT, MRI, or ultrasound based on the EASL guidelines^3^; (2) patients received TACE plus apatinib; (3) patients had Child–Pugh classification A or B; (4) patients had Eastern Cooperative Oncology Group (ECOG) scores of 0, 1, or 2; and (5) patients had complete follow-up data.

**Figure 1:**
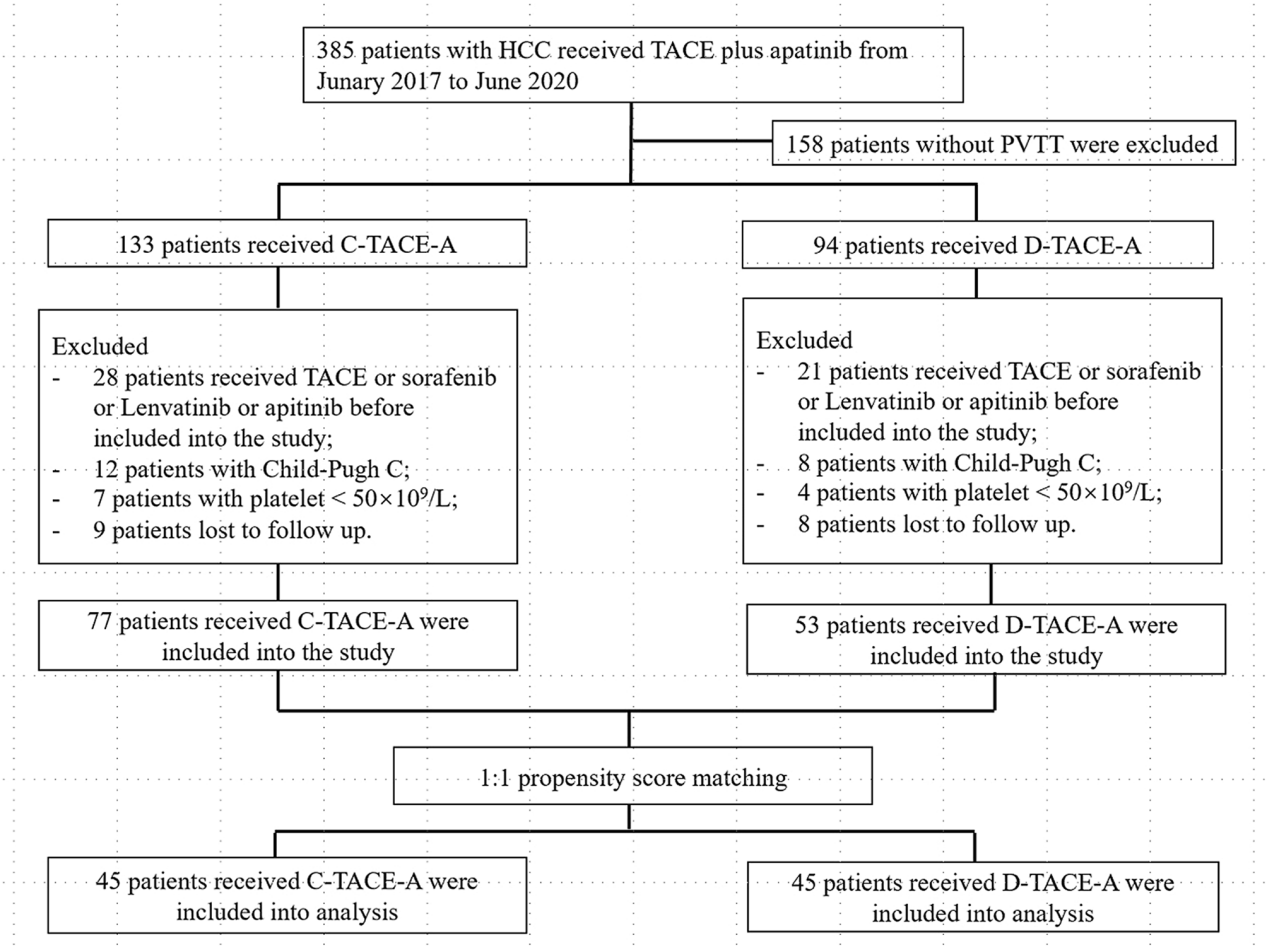
The flowchart of patient selection.

The exclusion criteria were as follows: (1) patients who received sorafenib, lenvatinib, or apatinib before being included in the study; (2) Patients with Child-Pugh C; (3) patients with platelet counts less than 50 × 10^9^/L; and (4) patients lost to follow-up (Fig. 1).

### Operation technique and drug administration

#### TACE protocol

TACE protocol was performed as the previous study stated^17^. After access to the femoral artery by the Seldinger technique under local anesthesia, a 5F catheter was positioned into the celiac artery. Celiac angiography was performed to relocate the tumor. Then, a 5F catheter or 3F microcatheter was inserted into the tumor artery according to the vascular anatomy.

For C-TACE, an emulsion mixed with lipiodol (10–20 mL) and epirubicin (5–20 mg) and subsequent 500–700 μm absorbable gelatin sponge particles (Alicon Medical C., Hangzhou, China) were injected into the tumor feeding artery for chemoembolization.

For D-TACE, CalliSphere beads (100–300 μm, Jiangsu Hengrui Medicine Co., Ltd., China) which was a special network of microsphere composed of polyvinyl alcohol backbone with hydroxyl groups and negatively charged sulfonate groups, which interact with positively charged anthracenes by means of ion exchange, hydrogen bonding, and embedding mechanisms carrying epirubicin (80 mg) were injected into the tumor feeding artery for chemoembolization.

#### Apatinib administration

Apatinib was administrated as the previous study stated^17^. In both groups, apatinib (Jiangsu Hengrui Medicine Co., Ltd., Jiangsu, China) was orally administered at an initial dose of 500 mg/day 3 days after the first TACE procedure. In the following TACE procedure, apatinib was started and suspended 3 days before and after each TACE procedure. The grading of adverse events related to apatinib was conducted according to the National Cancer Institute Common Terminology Criteria for Adverse Events (version 4.0). If serious adverse events (grade ≥ 3) were observed, a reduction to half of the dose was permitted until serious adverse events were eliminated. If these serious adverse events did not disappear after the dose reduction, the administration of apatinib would be interrupted temporarily. When these serious adverse events were eliminated, apatinib was orally retaken at a dose of 250 mg/day.

#### The endpoints and their definition

The primary endpoints of the study were overall survival (OS) and progression-free survival (PFS). OS was defined as the interval between the time of initial TACE and the patient’s death or the follow-up deadline. PFS was defined as the interval between the time of initial TACE and tumor progression, patient death, or the follow-up deadline based on the mRECIST. The secondary endpoints were the objective response rate (ORR) and disease control rate (DCR). The ORR was defined as the percent of the total number of patients who had a complete response (CR) and partial response (PR) after receiving TACE-A. The DCR was defined as the percent of the total number of patients who had a complete response (CR), partial response (PR), and stable disease (SD) after receiving TACE-A based on the mRECIST^18^.

#### Follow-up

Patients with PVTT who received TACE-A from January 2017 to June 2020 were retrospectively reviewed. The deadline for follow-up was June 2021. Patients were followed up every month after the initial TACE for three months and 2–3 months thereafter. Patients were asked to receive CT or MRI tests and laboratory tests at every follow-up. The CT or MRI images of patients at each follow-up were evaluated by two radiologists (one with 35 years of work experience and another with 28 years of work experience) and an interventional radiologist (with 16 years of work experience). When the tumor progressed (limited to the liver) during the follow-up, patients were recommended to receive another TACE to control the tumor.

### Statistical analysis

Age, ALT, leukocytes, lymphocytes, platelets, tumor size, sex, HBV infection, cirrhosis, TACE session, portal invasion, tumor number, extrahepatic metastases, AFP level, Child–Pugh classification, and ECOG score were included in the analysis. Continuous variables were compared by Student’s t-test or Mann–Whitney U test. Categorical variables were compared by the chi-square test or Fisher’s exact test. The survival curves were plotted by Kaplan–Meier curves and were compared by the log-rank test. A Cox proportional regression model was used to predict the variables that might influence the survival of patients. For subgroup analysis, an adjusted Cox proportional regression model was used, and the adjusted variables were age, ALT, leukocytes, lymphocytes, platelets, tumor size, sex, HBV infection, cirrhosis, tumor number, AFP level, Child–Pugh classification, and ECOG score.

Propensity score matching (PSM) was used in the study to reduce potential selective bias. All variables were included in the PSM analysis. Nearest neighbor matching (1:1 ratio) with an optimal caliper of 0.1 without replacement was conducted, and 45 pairs of patients were generated. A P value less than 0.05 was considered a statistically significant difference. All statistical analyses were conducted by SPSS 24.0 (IBM Corp, Armonk, NY, USA).

### Written informed consent to participate

Written informed consent was waived because this is a retrospective study.

### Guidelines for methods

This study was carried out in compliance with the Helsinki Declaration.

### Consent for publication

All authors approve it for publication.

## Results

### Patients’ characteristics

A total of 130 patients were included in the study. Among them, 77 patients received C-TACE-A, and 53 patients received D-TACE-A. There were 66 male patients and 11 female patients in the C-TACE-A group and 44 male patients and 9 female patients in the D-TACE-A group. In the C-TACE-A group, 33 patients had VP1–VP2 PVTT, and 44 patients had VP3–VP4 PVTT. In the D-TACE-A group, 23 patients had VP1–VP2 PVTT, and 30 patients had VP3–VP4 PVTT. The mean age in the C-TACE-A group was 51.3 years, and that in the D-TACE-A group was 53.1 years (Table 1).

**Table 1:** The baseline characteristics of patients before PSM and after PSM.

Characteristics	Before matching			After matching		
	C-TACE-A	D-TACE-A	P value	C-TACE-A	D-TACE-A	P value
Age (years)	51.3 ± 11.3	53.1 ± 9.4	0.335	51.3 ± 12.5	53.0 ± 9.9	0.478
ALT (U/L)	48.0 ± 34.1	61.8 ± 78.0	0.974	49.0 ± 35.4	50.7 ± 49.3	0.849
Leukocyte (10^9^/L)	5.5 ± 2.6	5.7 ± 2.0	0.718	5.4 ± 2.8	5.5 ± 2.0	0.837
Lymphocytes (10^9^/L)	1.3 ± 0.5	1.2 ± 0.5	0.221	1.2 ± 0.5	1.2 ± 0.5	0.896
Platelet (10^9^/L)	170.8 ± 87.7	163.8 ± 83.9	0.646	159.4 ± 85.4	163.7 ± 85.5	0.812
Tumor size (cm)	9.0 ± 4.3	10.9 ± 4.8	0.02	9.8 ± 4.5	10.3 ± 4.9	0.630
**Gender**			0.676			0.561
Male	66	44		39	37	
Female	11	9		6	8	
**HBV infection**			0.832			> 0.999
Yes	65	44		38	37	
No	12	9		7	8	
**Cirrhosis**			0.014			
Yes	58	29		30	28	0.660
No	19	24		15	17	
**TACE session**			0.188			0.803
Once	13	14		11	10	
Multiple	64	39		34	35	
**Portal invasion**			0.951			0.673
VP1–VP2	33	23		20	22	
VP3–VP4	44	30		25	23	
**Tumor number**			0.338			> 0.999
1	37	30		23	23	
≥ 2	40	23		22	22	
**Extrahepatic metastases**			0.807			> 0.999
Yes	39	28		23	23	
No	38	25		22	22	
**AFP level**			0.337			0.822
< 200 (ug/L)	28	15		14	15	
≥ 200 (ug/L)	49	38		31	30	
**Child–Pugh**			0.013			0.649
A	63	33		32	30	
B	14	20		13	15	
**ECOG**			0.975			0.963
0	27	18		18	18	
1	29	21		15	16	
2	21	14		12	11	

### Survival and tumor response before PSM

**Figure 2:**
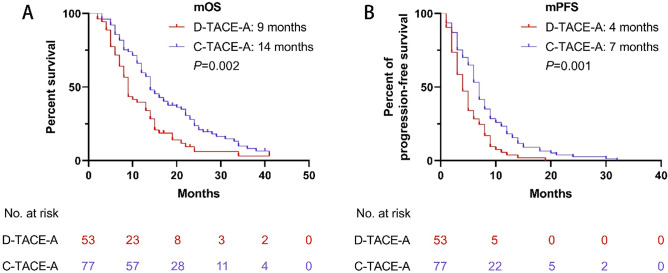
Kaplan–Meier curves of overall survival and progression-free survival in the patients before PSM. (**A**) Kaplan–Meier curve for overall survival; (**B**) Kaplan–Meier curve for progression-free survival.

The median OS (mOS) (14 months, 95% CI 11.7–16.3 months; vs 9 months, 95% CI 8–10 months; P = 0.002) and median PFS (mPFS) (7 months, 95% CI 6.1–7.9 months; vs 4 months, 95% CI 2.9–5.1 months; P = 0.001) in the C-TACE-A group was longer than the mOS and mPFS in the D-TACE-A group (Fig. 2).

The tumor response of 41 patients was PR, 16 patients had SD, and 20 patients had PD in the C-TACE group. The tumor response of 14 patients was PR, 13 patients had SD, and 26 patients had PD in the D-TACE-A group. The ORR (53.2% vs 26.4%; P = 0.002) and DCR (74% vs 50%; P = 0.007) in the C-TACE-A group were higher than those in the D-TACE-A group (Table 2).

**Table 2:** Analysis for tumor response at 3 months after patients receiving TACE-A.

Tumor response	Before matching			After matching		
	C-TACE-A	D-TACE-A	P value	C-TACE-A	D-TACE-A	P value
Partial response	41	14	–	23	13	–
Stable disease	16	13	–	6	12	–
Progressive disease	20	26	–	15	20	–
Objective response rate	53.2%	26.4%	0.002	48.9%	31.1%	0.021
Disease control rate	74%	50%	0.007	64.4%	57.8%	0.317

### Predictors for OS and PFS

The univariate analysis showed that the variables ALT (HR 1.003, 95% CI 1.001–1.006; P = 0.021), leukocytes (HR 1.141, 95% CI 1.056–1.233; P = 0.001), tumor size (HR 1.066, 95% CI 1.026–1.108; P = 0.001), TACE session (HR 0.127, 95% CI 0.076–0.212; P < 0.001), portal invasion (HR 2.729, 95% CI 1.859–4.007; P < 0.001), Child–Pugh (HR 3.187, 95% CI 2.104–4.825; P < 0.001), ECOG (1 vs 0: HR 4.908, 95% CI 2.969–8.111; P < 0.001; 2 vs 0: HR 11.710, 95% CI 6.737–20.352; P < 0.001), and treatment (HR 0.568, 95% CI 0.390–0.826; P = 0.003) might influence the OS of all patients. When these variables were included in multivariate analysis, the results showed that TACE session (HR 0.297, 95% CI 0.169–0.522; P < 0.001), portal invasion (HR 1.667, 95% CI 1.055–2.634; P = 0.029), Child–Pugh (HR 1.790, 95% CI 1.097–2.922; P = 0.020), ECOG (1 vs 0: HR 4.928, 95% CI 2.816–8.622; P < 0.001; 2 vs 0: HR 12.300, 95% CI 6.455–23.436; P < 0.001), and treatment (HR 0.387, 95% CI 0.253–0.591; P < 0.001) were independent predictors for OS (Table 3). For PFS, the univariate analysis showed that the variables ALT (HR 1.004, 95% CI 1.000–1.014; P = 0.027), leukocytes (HR 1.125; 95% CI 1.039–1.219; P = 0.004), tumor size (HR 1.055, 95% CI 1.016–1.095; P = 0.005), TACE session (HR 0.198, 95% CI 0.122–0.322; P < 0.001), portal invasion (HR 2.759, 95% CI 1.889–4.029; P < 0.001), Child–Pugh (HR 2.181; 95% CI 1.457–3.263; P < 0.001), ECOG (1 vs 0: HR 3.322, 95% CI 2.076–5.316; P < 0.001; 2 vs 0: HR 7.222, 95% CI 4.255–12.257; P < 0.001), and HR 0.559, 95% CI 0.389–0.804; P = 0.002) might influence tumor progression. In the multivariate analysis, TACE session (HR 0.397, 95% CI 0.229–2.869; P = 0.001), portal invasion (HR 1.855, 95% CI 1.200–2.869; P = 0.005), ECOG (1 vs 0: HR 3.083, 95% CI 1.850–5.135; P < 0.001; 2 vs 0: HR 6.976, 95% CI 3.731–113.044; P < 0.001), and treatment (HR 0.402, 95% CI 0.270–0.600; P < 0.001) were independent predictors for PFS (Table 4).

**Table 3:** Univariate regression analysis and multivariate regression analysis for OS before PSM.

Characteristics	Univariate analysis		Multivariate analysis	
	HR (95% CI)	P value	HR (95% CI)	P value
Age (years)	1.003 (0.986, 1.020)	0.733		
ALT (U/L)	1.003 (1.001, 1.006)	0.021	0.998 (0.994, 1.001)	0.230
Leukocyte (10^9^/L)	1.141 (1.056, 1.233)	0.001	1.073 (0.989, 1.165)	0.090
Lymphocytes (10^9^/L)	0.741 (0.515, 1.066)	0.106		
Platelet (10^9^/L)	1.002 (1.000, 1.004)	0.118		
Tumor size (cm)	1.066 (1.026, 1.108)	0.001	1.018 (0.976, 1.062)	0.415
**Gender**		0.089		
Male	1			
Female	0.632 (0.372, 1.073)			
**HBV infection**		0.907		
Yes	1			
No	1.030 (0.629, 1.685)			
**Cirrhosis**		0.522		
Yes	1			
No	1.135 (0.770, 1.672)			
**TACE session**		< 0.001		< 0.001
Once	1		1	
Multiple	0.127 (0.076, 0.212)		0.297 (0.169, 0.522)	
**Portal invasion**		< 0.001		0.029
VP1–VP2	1		1	
VP3–VP4	2.729 (1.859, 4.007)		1.667 (1.055, 2.634)	
**Tumor number**		0.688		
1	1			
≥ 2	1.077 (0.750, 1.548)			
**Extrahepatic metastases**		0.100		
Yes	1			
No	0.736 (0.510, 1.061)			
**AFP level**		0.083		
< 200 (ug/L)	1			
≥ 200 (ug/L)	1.410 (0.957, 2.077)			
**Child–Pugh**		< 0.001		0.020
A	1		1	
B	3.187 (2.104, 4.825)		1.790 (1.097, 2.922)	
**ECOG**				
0	1		1	
1	4.908 (2.969, 8.111)	< 0.001	4.928 (2.816, 8.622)	< 0.001
2	11.710 (6.737, 20.352)	< 0.001	12.300 (6.455, 23.436)	< 0.001
**Treatment**		0.003		< 0.001
D-TACE-A	1		1	
C-TACE-A	0.568 (0.390, 0.826)		0.387 (0.253, 0.591)

**Table 4:** Univariate regression analysis and multivariate regression analysis for PFS before PSM.

Characteristics	Univariate analysis		Multivariate analysis	
	HR (95% CI)	P value	HR (95% CI)	P value
Age (years)	0.998 (0.983, 1.014)	0.834		
ALT (U/L)	1.004 (1.000, 1.007)	0.027	0.997 (0.993, 1.001)	0.137
Leukocyte (10^9^/L)	1.125 (1.039, 1.219)	0.004	1.070 (0.984, 1.165)	0.114
Lymphocytes (10^9^/L)	0.742 (0.528, 1.043)	0.086		
Platelet (10^9^/L)	1.001 (0.999, 1.003)	0.201		
Tumor size (cm)	1.055 (1.016, 1.095)	0.005	1.005 (0.966, 1.047)	0.800
**Gender**		0.187		
Male	1			
Female	0.717 (0.437, 1.176)			
**HBV infection**		0.418		
Yes	1			
No	0.819 (0.504, 1.329)			
**Cirrhosis**		0.429		
Yes	1			
No	1.160 (0.803, 1.675)			
**TACE session**		< 0.001		0.001
Once	1		1	
Multiple	0.198 (0.122, 0.322)		0.397 (0.229, 2.869)	
**Portal invasion**		< 0.001		0.005
VP1–VP2	1		1	
VP3–VP4	2.759 (1.889, 4.029)		1.855 (1.200, 2.869)	
**Tumor number**		0.636		
1	1			
≥ 2	0.919 (0.646, 1.306)			
**Extrahepatic metastases**		0.084		
Yes	1			
No	0.733 (0.514, 1.043)			
**AFP level**		0.074		
< 200 (ug/L)	1			
≥ 200 (ug/L)	1.403 (0.968, 2.034)			
**Child–Pugh**		< 0.001		0.747
A	1		1	
B	2.181 (1.457, 3.263)		1.081 (0.672, 1.740)	
**ECOG**				
0	1		1	
1	3.322 (2.076, 5.316)	< 0.001	3.083 (1.850, 5.135)	< 0.001
2	7.222 (4.255, 12.257)	< 0.001	6.976 (3.731, 13.044)	< 0.001
**Treatment**		0.002		
D-TACE-A	1		1	
C-TACE-A	0.559 (0.389, 0.804)		0.402 (0.270, 0.600)	< 0.001

### Subgroup analysis

The adjusted Cox proportional regression model showed that patients with VP1–VP2 (HR 0.156, 95% CI 0.063–0.389; P < 0.001), VP3–VP4 (HR 0.439, 95% CI 0.227–0.851; P = 0.015), without extrahepatic metastases (HR 0.252, 95% CI 0.116–0.545; P < 0.001), with one TACE session (HR 0.194, 95% CI 0.054–0.700; P = 0.012), and with multiple TACE sessions (HR 0.343, 95% CI 0.195–0.602; P < 0.001) who received C-TACE-A had a lower risk of death than patients who received D-TACE-A (Table 5). However, for PFS, only patients with VP1–VP2 (HR 0.155, 95% CI 0.065–0.370; P < 0.001), without extrahepatic metastases (HR 0.334, 95% CI 0.165–0.673; P = 0.002), and with multiple TACE sessions (HR 0.431, 95% CI 0.265–0.702; P = 0.001) who received C-TACE-A had a lower tumor progression risk than patients who received D-TACE-A (Table 6).

**Table 5:** Adjusted Cox proportional risk analysis of OS for subgroup analysis before PSM.

Characteristics	Crude analysis		Adjusted analysis	
	HR (95% CI)	P value	HR (95% CI)	P value
**Portal invasion**				
VP1–VP2		0.026		< 0.001
D-TACE-A	1		1	
C-TACE-A	0.513 (0.285, 0.923)		0.156 (0.063, 0.389)	
VP3–VP4		0.011		0.015
D-TACE-A	1		1	
C-TACE-A	0.525 (0.319, 0.864)		0.439 (0.227, 0.851)	
**Extrahepatic metastases**				
With extrahepatic metastases		0.191		0.086
D-TACE-A	1		1	
C-TACE-A	0.709 (0.423, 1.187)		0.572 (0.303, 1.045)	
Without extrahepatic metastases		0.004		< 0.001
D-TACE-A	1			
C-TACE-A	0.440 (0.253, 0.765)		0.252 (0.116, 0.545)	
**TACE session**				0.012
Once		0.244		
D-TACE-A	1		1	
C-TACE-A	0.624 (0.2882, 1.380)		0.194 (0.054, 0.700)	
Multiple		0.012		< 0.001
D-TACE-A	1		1	
C-TACE-A	0.573 (0.372, 0.883)		0.343 (0.195, 0.602)	

Adjusted for age, ALT, leukocyte, lymphocytes, platelet, tumor size, gender, HBV infection, cirrhosis, TACE session, tumor number, AFP level, Child–Pugh, ECOG.

**Table 6:** Adjusted Cox proportional risk analysis of PFS for subgroup analysis before PSM.

Characteristics	Crude analysis		Adjusted analysis	
	HR (95% CI)	P value	HR (95% CI)	P value
**VP1–VP2**		0.003		< 0.001
D-TACE-A	1		1	
C-TACE-A	0.424 (0.242, 0.744)		0.155 (0.065, 0.370)	
**VP3–VP4**		0.074		0.278
D-TACE-A	1		1	
C-TACE-A	0.649 (0.405, 1.042)		0.722 (0.401, 1.300)	
**With extrahepatic metastases**		0.115		0.077
D-TACE-A	1		1	
C-TACE-A	0.672 (0.410, 1.102)		0.585 (0.323, 1.060)	
**Without extrahepatic metastases**		0.009		0.002
D-TACE-A	1		1	
C-TACE-A	0.490 (0.287, 0.838)		0.334 (0.165, 0.673)	
**TACE session**				
Once		0.287		0.266
D-TACE-A	1		1	
C-TACE-A	0.640 (0.282, 1.454)		0.504 (0.151, 1.686)	
Multiple		0.006		0.001
D-TACE-A	1		1	
C-TACE-A	0.559 (0.370, 0.846)		0.431 (0.265, 0.702)	

Adjusted for age, ALT, leukocyte, lymphocytes, platelet, tumor size, gender, HBV infection, cirrhosis, tumor number, AFP level, Child–Pugh, ECOG.

### Suvival and tumor response after PSM

**Figure 3:**
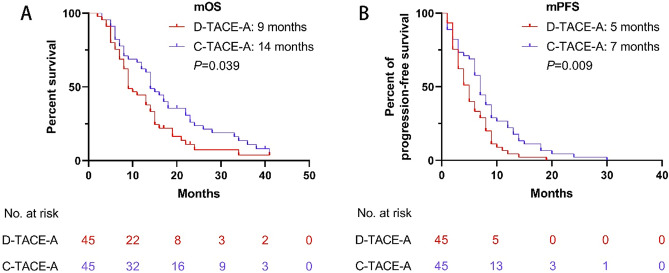
Kaplan–Meier curves of overall survival and progression-free survival in the patients after PSM. (**A**) Kaplan–Meier curve for overall survival; (**B**) Kaplan–Meier curve for progression-free survival.

The mOS (14 months, 95% CI 11.2–16.8 months) and mPFS (7 months, 95% CI 5.9–8.1 months) in the C-TACE-A group were longer than the mOS (9 months, 95% CI 6.5–11.5 months; P = 0.039) and mPFS (5 months, 95% CI 3.7–6.3 months; P = 0.009) in the D-TACE-A group (Fig. 3). The ORR (48.9%) in the C-TACE-A group was higher than the ORR (31.1%; P = 0.021) in the D-TACE-A group. However, there was no statistically significant difference in DCR (64.4% vs 57.8%; P = 0.317) between the two groups (Table 2).

### Safety

The adverse events of patients with TACE-A were reported and evaluated. For all grades of adverse events, there was no statistically significant difference in fever, fatigue, nausea, vomiting, diarrhea, hand-foot syndrome, hypertension, proteinuria, or hyperbilirubinemia between the C-TACE-A group and D-TACE-A group (all P > 0.05). However, the adverse event of pain was significantly higher in the C-TACE-A group than that in the D-TACE-A group (34 patients vs 9 patients; P = 0.001). For serious adverse events (grades III and IV), there was no statistically significant difference in any adverse events reported in the study (all P > 0.05) (Table 7).

**Table 7:** The adverse events of patients with TACE-A before PSM.

Adverse events	All grades			III and IV grades		
	C-TACE-A (N/%)	D-TACE-A (N/%)	P value	C-TACE-A (N/%)	D-TACE-A (N/%)	P value
Fever	39 (50.6)	32 (60.4)	0.087	5 (6.5)	5 (9.4)	0.777
Fatigue	24 (31.2)	16 (30.2)	0.905	2 (2.6)	1 (1.9)	0.789
Pain	34 (44.2)	9 (17.0)	0.001	8 (10.4)	2 (3.8)	0.198
Nausea	21 (27.3)	16 (30.2)	0.408	3 (3.9)	1 (1.9)	0.892
Vomiting	15 (19.5)	12 (22.6)	0.662	1 (2.3)	2 (3.8)	0.567
Diarrhea	11 (14.3)	12 (22.6)	0.220	0 (0)	1 (1.9)	0.408
Hand-foot syndrome	16 (20.8)	14 (26.4)	0.454	3 (3.9)	3 (5.6)	0.687
Hypertension	25 (32.5)	20 (37.7)	0.535	5 (6.5)	4 (7.5)	0.817
Proteinuria	12 (15.6)	11 (20.8)	0.448	1 (2.3)	0 (0)	> 0.999
Hyperbilirubinemia	5 (6.5)	5 (9.4)	0.777	0 (0)	0 (0)	> 0.999

## Discussion

HCC patients with PVTT have a dismal prognosis. Recently, some studies have shown that HCC patients could obtain more survival benefits from C-TACE plus apatinib than TACE alone. However, the efficacy and safety of HCC patients with PVTT who received D-TACE-A have not been reported. Thus, the study was conducted to compare the efficacy and safety of C-TACE-A and D-TACE-A in the treatment of HCC patients with PVTT.

The main findings of the study were that HCC patients with PVTT could obtain more survival benefits from C-TACE-A than D-TACE-A, especially patients with VP1–VP2, without extrahepatic metastases, and with multiple TACE sessions. The ORR at three months after the initial TACE of the C-TACE-A group was higher than that of the D-TACE-A group. The reason for the results might be that the embolic agent of lipiodol has strong fluidity, which can flow into the tumor thrombus and stay in the tumor thrombus, achieving a good embolization effect. A previous study showed that the mOS of HCC patients receiving C-TACE-A was 12 months, which was less than the mOS from the current study^19^. The DCR (59%) in that study was also lower than the DCR (74%) in the current study. The reason might be that they included more patients with high stage PVTT than the patients included in the current study because the PVTT stage could influence the survival of patients by multivariate analysis. Another study included 19 HCC patients with PVTT who received D-TACE-A. The results showed that the mOS, mPFS, ORR, and DCR at 1 month after the initial TACE of the 19 patients were 11.9 months, 8.1 months, 63.15%, and 84.21%, respectively, which were all higher than the mOS, ORR, and DCR in the current study^20^. The ORR and DCR of that study were higher than those in the current study, possibly because they evaluated the 1st-month tumor response after the initial TACE, but the current study evaluated the 3rd-month tumor response after the initial TACE. The samples of previous studies and the current study were not larger, which might lead to the heterogeneity of patients being large and the different survival results of different studies. Thus, a larger sample study is needed to confirm the results of the current study.

Multivariate Cox regression analysis is common used to exclude the influence of potential factors on the results. In the study, we conducted the univariate regression analysis and the factors with P value less than 0.05 in the univariate regression analysis were included into multivariate regression analysis to reduce the rate of collinearity. The multivariate regression analysis in the study showed that patients with C-TACE-A had a lower all-cause mortality risk and tumor progression risk than patients with D-TACE-A, which showed that HCC patients with PVTT could still obtain more survival benefits from C-TACE-A than D-TACE-A after excluding potential influencing factors.

The results of previous studies and the current study showed that TACE sessions, PVTT stage, and extrahepatic metastases could influence the survival of patients^21–26^. Thus, subgroup analysis was conducted based on the factors. After adjusting for relative variables, the results showed that patients with VP1–VP2 PVTT, without extrahepatic metastases, and with multiple TACE sessions could obtain more survival benefits from C-TACE-A than D-TACE-A. The results might be used for clinics to select more suitable treatments for these patients.

This study reported all-grade adverse events and serious adverse events of patients with C-TACE-A and D-TACE-A. The results showed that patients in the C-TACE-A group had a higher pain risk than patients in the D-TACE-A group. The result was consistent with previous studies^3,16,27^. For serious adverse events, there was no statistically significant difference in adverse events between the two groups, which should be because C-TACE-A was as safe as D-TACE-A in the treatment of HCC patients with PVTT because grade I and II adverse events could be cured well by symptomatic treatment.

There are some limitations existing in the study. First, although PSM was conducted in the study, selection bias was inevitable because the study was retrospective. Second, the sample of the study was not large, which might influence the evidence strength of the results. Thus, a larger sample study or prospective research is needed to confirm this hypothesis.

## Conclusion

This study showed that hepatocellular carcinoma patients with portal vein tumor thrombus could obtain more survival benefits from conventional transarterial chemoembolization plus apatinib than drug-eluting bead transarterial chemoembolization plus apatinib, especially for patients with VP1–VP2 PVTT, without extrahepatic metastases, with multiple TACE session treatments. The results of the study might provide new evidence for clinics to select suitable treatments for advanced hepatocellular carcinoma patients with portal vein tumor thrombus.

## Data Availability

The data can be available from the correspondence authors upon reasonable request.
